# Comparing Outcomes in Asymptomatic and Symptomatic Atrial Fibrillation: A Systematic Review and Meta-Analysis of 81,462 Patients

**DOI:** 10.3390/jcm10173979

**Published:** 2021-09-02

**Authors:** Daria Sgreccia, Marcella Manicardi, Vincenzo Livio Malavasi, Marco Vitolo, Anna Chiara Valenti, Marco Proietti, Gregory Y. H. Lip, Giuseppe Boriani

**Affiliations:** 1 Cardiology Division, Department of Biomedical, Metabolic and Neural Sciences, University of Modena and Reggio Emilia, Policlinico di Modena, 41124 Modena, Italy; daria.sgreccia@gmail.com (D.S.); marcella.manicardi@gmail.com (M.M.); nanni.malavasi@gmail.com (V.L.M.); marco.vitolo@unimore.it (M.V.); annachiara.valenti@gmail.com (A.C.V.); 2 Clinical and Experimental Medicine PhD Program, University of Modena and Reggio Emilia, 41124 Modena, Italy; 3 Liverpool Centre for Cardiovascular Science, University of Liverpool and Liverpool Heart & Chest Hospital, Liverpool L14 3PE, UK; marco.proietti@unimi.it (M.P.); gregory.lip@liverpool.ac.uk (G.Y.H.L.); 4 Geriatric Unit, IRCCS Istituti Clinici Scientifici Maugeri, 20138 Milan, Italy; 5 Department of Clinical Sciences and Community Health, University of Milan, 20122 Milan, Italy; 6 Department of Clinical Medicine, Aalborg University, DK-9100 Aalborg, Denmark

**Keywords:** atrial fibrillation, symptoms, outcomes, stroke, mortality, meta-analysis

## Abstract

Background: In atrial fibrillation (AF) patients, the presence of symptoms can guide the decision between rate or rhythm control therapy, but it is still unclear if AF-related outcomes are determined by symptomatic status of their clinical presentation. Methods: We performed a systematic review and metanalysis following the PRISMA recommendations on available studies that compared asymptomatic to symptomatic AF reporting data on all-cause mortality, cardiovascular death, and thromboembolic events (TEs). We included studies with a total number of patients enrolled equal to or greater than 200, with a minimum follow-up period of six months. Results: From the initial 5476 results retrieved after duplicates’ removal, a total of 10 studies were selected. Overall, 81,462 patients were included, of which 21,007 (26%) were asymptomatic, while 60,455 (74%) were symptomatic. No differences were found between symptomatic and asymptomatic patients regarding the risks of all-cause death (odds ratio (OR) 1.03, 95% confidence interval (CI) 0.81–1.32), and cardiovascular death (OR 0.87, 95% CI 0.54–1.39). No differences between symptomatic and asymptomatic groups were evident for stroke (OR 1.22, 95% CI 0.77–1.93) and stroke/TE (OR 1.06, 95% CI 0.86–1.31) risks. Conclusions: Mortality and stroke/TE events in AF patients were unrelated to symptomatic status of their clinical presentation. Adoption of management strategies in AF patients should not be based on symptomatic clinical status.

## 1. Introduction

Atrial fibrillation (AF) is the most common sustained arrhythmia in adults and approximately one-third of AF patients are asymptomatic [1]. In such cases, AF is frequently detected during clinical screening in different settings (i.e., pre-operative assessments, cryptogenic stroke, continuous rhythm monitoring through an implanted device) [2,3,4,5,6,7,8,9]. Patients with AF are at increased risk of stroke and thromboembolic events (TEs), heart failure, cognitive impairment, and death [1,10,11,12,13]. As the diagnosis of AF may be delayed in asymptomatic patients, TE may be the first clinical presentation in these patients. 

Systematic or opportunistic screening for silent AF has thus been proposed and performed in different settings [14,15,16]. The background supporting the value of AF screening is the possibility to discover patients with unknown AF with a risk profile of AF-related mortality and morbidity complications such as stroke/TE, in order to appropriately institute oral anticoagulant (OAC) therapy.

It is not completely understood why some patients with AF develop symptoms while other patients are totally asymptomatic and to what extent the clinical status may be associated with adverse outcomes [17]. Indeed, patient characteristics, such as concomitant cardiac conditions or somatic and psychological factors, can contribute to the complex relationship between AF and symptoms [18]. In older patients, symptoms could decrease or disappear with longer arrhythmia duration [18,19].

Although the presence of symptoms may guide the decision between rate or rhythm control therapy, it is unclear if asymptomatic or symptomatic clinical presentations are related to outcomes. The evidence is conflicting as several studies have reported that asymptomatic patients show similar risk for TE, in particular ischemic stroke and death, compared with symptomatic patients [20,21,22,23]. On the other hand, other studies have reported an association between mortality, TE, and silent AF [24,25].

Therefore, we performed a systematic review and meta-analysis aimed at attesting the hypothesis that asymptomatic AF patients have a higher risk of all-cause mortality, cardiovascular death, and thromboembolic events compared with symptomatic AF patients.

## 2. Materials and Methods

This systematic review and meta-analysis was conducted following the preferred reporting items for systematic reviews and meta-analysis (PRISMA) recommendations (http://www.prisma-statement.org, accessed on 1 April 2021). We performed an extensive search in three major medical literature databases: PubMed, Embase, and Cochrane, for available studies, published in English, that have compared asymptomatic to symptomatic AF reporting data on all-cause mortality, cardiovascular death, and TE events or stroke. The search strategy used a combination of the following terms or their synonyms: (asymptomatic OR symptomatic) AND atrial fibrillation AND outcome; (asymptomatic OR symptomatic) AND atrial fibrillation AND (outcome OR death OR mortality OR stroke OR thromboembolism OR embolism). The whole syntax is shown in the Supplementary Materials (Table S1).

### 2.1. Study Selection 

Two investigators (D.S. and M.M.) independently screened records for eligibility based on titles and abstracts. Full texts of articles deemed potentially eligible were then screened independently by each investigator for final inclusion. Disagreement was resolved via consensus and discussion or, if necessary, through consultation with a third reviewer (V.L.M.). Finally, articles that fulfilled the following inclusion criteria were selected for the meta-analysis: (i) studies published in English; (ii) observational studies or randomized controlled trials; (iii) total number of patients enrolled equal to or greater than 200; (iv) studies on AF patients divided into asymptomatic and symptomatic groups; and (v) minimum follow-up period of six months.

### 2.2. Data Extraction and Management

Data extraction was performed by two investigators (D.S. and M.M.) independently, and disagreements were resolved by a third investigator (V.L.M.). When available, the following data were extracted from each study: number of patients enrolled, mean or median age, sex, diabetes, coronary artery disease, heart failure, thromboembolic risk, antithrombotic treatment, beta blockers therapy, antiarrhythmic drugs, catheter ablation intervention, follow-up time, all-cause death, cardiovascular death, ischemic stroke, and TE (considered in the analysis as systemic TE/stroke). For this meta-analysis, the outcomes of interest were as follows: (i) all-cause death; (ii) CV-death; (iii), TE/stroke, and (iv) stroke. 

### 2.3. Quality Assessment

Two investigators (D.S. and M.M.) independently evaluated all the studies to assess the risk of bias. The Newcastle-Ottawa scale was used to assess the methodological qualities of non-randomized studies [26]. Studies were considered to be of high quality when scoring ≥ 5. The risk of bias of randomized clinical trials results was evaluated following Version 2 of the Cochrane risk-of-bias tool for randomized trials (RoB2).

### 2.4. Statistical Analysis

Continuous variables are reported as mean or median, and categorical variables as number and percentage. The Mantel-Haenszel random-effects model was used to determine the pooled odds ratio (OR) and the corresponding 95% confidence interval (CI) for outcomes of interest. For each outcome of interest, the summary result of the meta-analysis and results of individual studies were shown using forest plots.

Heterogeneity among studies was assessed using the I2 statistic index. Thresholds for the interpretation of I2 were low heterogeneity if I2 < 25%, moderate if I2 between 25 and 75%, and high if I2 > 75%. To assess the influence of each single study on heterogeneity, we performed a sensitivity analysis with a “leave-one-out” approach if I2 was >25% (moderate or high heterogeneity). To further investigate the reasons for the heterogeneity, we perform a meta-regression analysis, using the variables specified in original papers (type of study, number of males, diabetes, coronary artery disease, heart failure, use of OAC) as a moderator and comparing the relative I2 with that of the main analysis (all-cause death and stroke or systemic embolism). The variables were forced into the model one at a time. Publication bias was assessed by visual inspection of funnel plot and by Egger’s test [27].

All statistical analyses were performed using Revman 5 (Review Manager (RevMan) Version 5.4. The Cochrane Collaboration, London, UK). JAMOVI 1.6 (https://www.jamovi.org, accessed on 1 April 2021) with module MAJOR v. 1.2, a graphic user interface for R v. 4 (https://www.r-project.org/, accessed on 1 April 2021) and package “meta-for” 1.4, was used to perform the meta-regression [28].

## 3. Results

**Figure 1:**
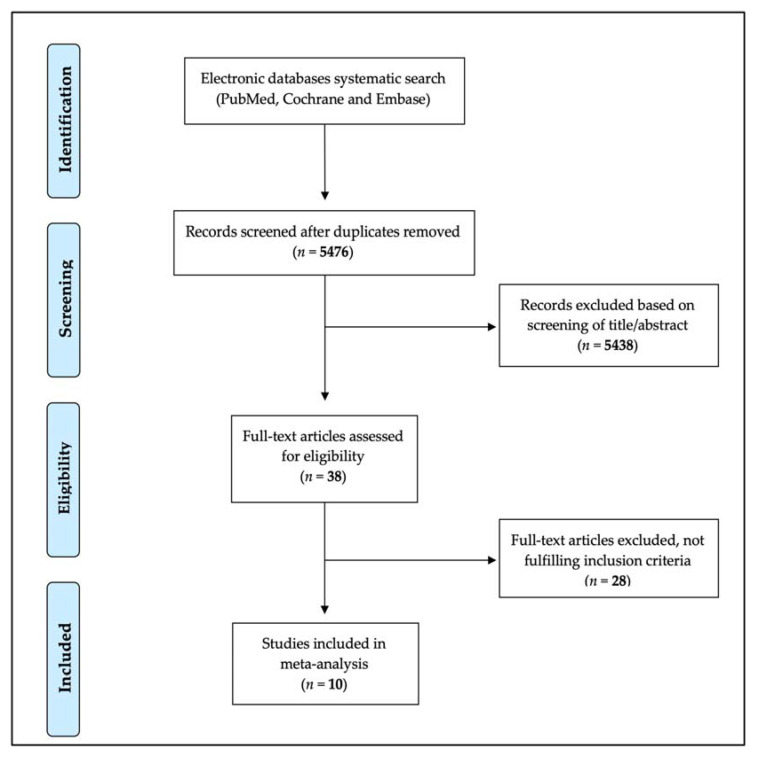
Study selection process (PRISMA Flow Diagram).

A systematic search of electronic databases identified a total of 5476 articles, after removing duplicates. Of these, 5438 were excluded based on title and abstract. The remaining 38 were evaluated through full text revision. Finally, 10 articles fulfilled the inclusion criteria and were included in the analysis (Figure 1) [17,19,20,21,22,23,24,29,30,31].

Study design and baseline characteristics of the included studies are summarized in Table 1. Of the 10 studies included, 2 were retrospective observational studies [23,28], 6 were observational prospective studies [20,21,22,24,30,31], and 2 were derived from clinical randomized trials [17,19]. Studies ranged from 2005 to 2021, while the sample size ranged from 334 to 52,032 patients.

Overall, 81,462 patients were included in this meta-analysis, and of these, 21,007 (26%) were asymptomatic while 60,455 (74%) were symptomatic. Data from the Euro Heart Survey on Atrial Fibrillation [31] were presented according to symptoms at baseline and the development of symptoms after one-year follow-up. Asymptomatic patients at baseline were classified into still asymptomatic (AA) or symptomatic (AS) at one year follow-up; similarly, symptomatic patients at baseline were classified into still symptomatic (SS) or asymptomatic (SA) at one-year follow-up, with clinical outcomes merged based on baseline presentation, while clinical characteristics were reported separately [31]. Data on thromboembolic risk and antithrombotic treatments are shown in Table 2. Antiarrhythmics treatments are shown in Table S2.

Definitions of TE were different between studies; Boriani et al. [24] and Gibbs et al. [20] defined TE as the combination of non-hemorrhagic stroke (ischemic stroke and stroke of unknown origin) and systemic thromboembolic event, while others [30,31] separately reported TE (defined as peripheral/arterial or pulmonary embolism) and stroke. The definition of AF was considered as the finding in an ECG or in a Holter recording of the arrythmia for all of the studies considered. Potpara et al. [21] considered in their analysis patients with first diagnosis of non-valvular AF, whereas Senoo et al. [23] considered only patients with paroxysmal atrial fibrillation defined as (1) sinus rhythm on ECG and previous diagnosis of paroxysmal AF by referring physicians; (2) symptomatic AF on ECG at the initial visit and duration of AF estimated as <7 days according to symptoms or ECG recordings; and (3) asymptomatic AF on ECG at the initial visit and no AF 1 week prior. The incidence of the type of AF in asymptomatic and symptomatic patients for each study is shown in Table S3.

The evaluation of symptoms was different in the studies considered, but comparable, and appeared sufficiently suitable and complete to identify the presence or absence of AF-related symptoms. Some studies [22,24,30] used the EHRA score, two studies used a symptom checklist [17,19], while the remaining studies [20,21,23,29,31] evaluated symptoms according to physician’s clinical judgment. An overview of the tools used to identify AF-related symptoms is shown in detail in Table S4.

Age differences for symptomatic and asymptomatic AF patients are shown in Figure S1. Seven studies [17,19,21,23,29,30,31] were included for the analyses, and no significant differences were found between symptomatic and asymptomatic AF patients (standardized mean difference 0.06; 95% CI −0.05–0.17). The I^2^ statistic was 83%, indicating high heterogeneity. Additionally, no significant differences in stroke risk profile were found among the studies detailed [17,24,29,30,31] (standardized mean difference CHA_2_DS_2_VASc = −0.05, 95% CI −0.27–0.17, I^2^ = 96%; CHADS_2_ = −0.03; 95% CI −0.22–0.16; I^2^ = 91%) (Figure S2 panel A and B).

### 3.1. All-Cause and Cardiovascular Mortality

**Figure 2:**
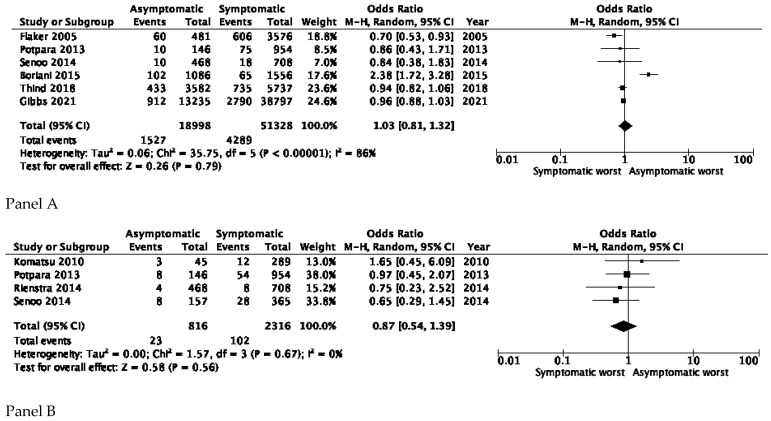
All cause and cardiovascular mortality. Panel (**A**) All-cause death in asymptomatic and symptomatic AF patients. Panel (**B**) Cardiovascular death in asymptomatic and symptomatic AF patients.

All-cause mortality was available for six studies [19,20,21,22,23,24]. No significant differences were found between symptomatic and asymptomatic patients (OR 1.03, 95% CI 0.81–1.32); however, there was considerable heterogeneity (I^2^ = 86%) (Figure 2, Panel A).

Four studies [17,21,23,29] reported data on cardiovascular death, with no significant differences between symptomatic and asymptomatic AF (OR 0.87, 95% CI 0.54–1.39) (Figure 2B). The value of I^2^ was 0%, indicating homogeneity in the effect (Figure 2, Panel B).

### 3.2. Stroke and Thromboembolic Events 

**Figure 3:**
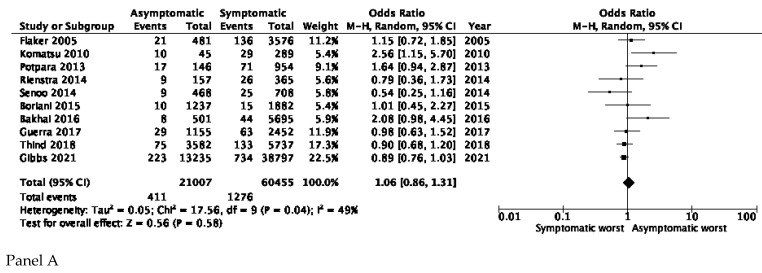

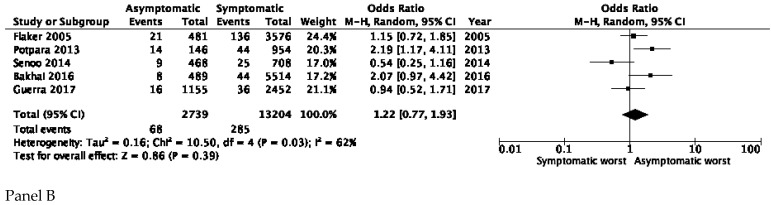
Stroke and thromboembolic events. Panel (**A**) Thromboembolic events/stroke in asymptomatic and symptomatic AF patients. Panel (**B**) Stroke in asymptomatic and symptomatic AF patients.

The endpoint of TE/stroke was available for all of the studies [17,19,20,21,22,23,24,29,30,31] and the analysis showed no significant differences between asymptomatic and symptomatic AF patients (OR 1.06, 95% CI 0.86–1.31) with moderate heterogeneity (I^2^ = 49%) (Figure 3, Panel A). Five studies [19,21,23,30,31] reported data on non-haemorrhagic stroke; the overall pooled estimate showed no significant differences between asymptomatic and symptomatic AF patients (OR 1.22, 95% CI 0.77–1.93) with moderate heterogeneity (I^2^ = 62%) (Figure 3, Panel B).

### 3.3. Risk of Bias of Included Studies 

The Newcastle–Ottawa scale was used for the critical appraisal of non-randomized studies (Table S5). All studies performed well in terms of selection of population, which consisted of AF patients with different clinical status (asymptomatic and symptomatic). In most of the studies considered, there was a percentage of patients with prior stroke/TE, which potentially confers a risk of bias when assessing the stroke/TE outcome in the population. Risk factors for death and TE were considered by all studies, and nine (out of ten) studies [17,19,20,21,22,23,24,29,31] made adjustments for more than one risk factor (i.e., age, sex, diabetes, CHADS_2_ score). Both exposure and outcome were derived from secure records for all subjects included in the studies (i.e., data extracted from electronic case report file, codes from databases of medical records, cerebral TE diagnoses made on imaging records as computed tomographic examination or magnetic resonance imaging). The two randomized clinical trials [17,19] were considered at low risk of bias using the Cochrane RoB 2 tool.

The visual inspection of the funnel plots (Figure S3) and Egger’s tests did not show significant publication bias for each outcome considered (all-cause death *p* = 0.728; CV death *p* = 0.666; stroke and/or any thromboembolism *p* = 0.146).

### 3.4. Sensitivity Analysis 

Regarding all-cause death, the sensitivity analysis showed that the study by Boriani et al. [24] was the most relevant contributor to the heterogeneity (Table S6). When considering the other outcomes of interest, the test for overall effect and heterogeneity did not substantially change along the sensitivity analyses (Table S6).

### 3.5. Inspection of Heterogeneity

In order to address potential causes of heterogeneity, we performed meta-regressions for all the outcomes considered. Meta-regression on all-cause death did not add further information as the range of I^2^ was 88–91% for all moderators, while the main analysis had an I^2^ equal to 86% (Table S7). With regard to stroke or systemic embolism, the meta-regression showed that type of study (retrospective vs. prospective), diabetes mellitus, and OAC did not change the direction of analysis, but the heterogeneity decreased from 49% to 28% for coronary artery disease and from 49% to 12% for heart failure (Table S8).

## 4. Discussion

### 4.1. Major Findings

The main findings of our systematic review and meta-analysis are as follows: (i) symptomatic and asymptomatic AF patients have comparable likelihood for all-cause death and cardiovascular mortality; (ii) TE events had a similar incidence rate between the two groups; and (iii) in AF patients, clinical outcomes appear to be non-dependent on the presence or absence of symptoms at clinical presentation.

Our study includes a substantially much larger number of patients as compared with a previous meta-analysis [32], which had 10,308 patients, where asymptomatic and symptomatic AF patients showed similar outcomes (OR for all-cause death: 1.38, 95% CI 0.87–2.17; OR for cardiovascular death 0.85, 95% CI 0.53–1.36; OR for TE 1.39, 95% CI 0.72–2.68).

Moreover, the CHA2DS2VASc score was analysed in our study, and this may be of value for interpreting our data, in view of the fact that this score is the widely accepted reference for decision making. The heterogeneity found in our analysis suggests that substantial differences in terms of mortality and TE risk occurred among the ten studies included. Data from the EORP-AF Pilot Registry [24] showed that asymptomatic AF patients had a higher occurrence of all-cause death compared with symptomatic patients (9.4% vs. 4.2%, respectively; *p* < 0.0001), but this was not confirmed in the other studies considered.

The high heterogeneity found analysing all-cause death is largely due to the study of Boriani et al. [24]. Indeed, in the sensitivity analysis, removing this study, the heterogeneity falls to 10%, reaching a borderline statistical significance (Table S6; *p* = 0.05 for overall effect). This finding could be, at least partly, explained by a threefold higher prevalence of permanent AF; by older age of the asymptomatic patients; and, primarily, by the high prevalence of ischemic heart disease reported in the asymptomatic group of this study (Table 1). However, the finding of a higher risk of death associated with asymptomatic AF is not unique of this study, as a report from the Mayo Clinic [25] showed the same association between asymptomatic AF and all-cause mortality, even after adjustment for CHA2DS2-VASc score and age. Unfortunately, we could not include this study from the Mayo Clinic [25], as only a time-dependent analysis was reported and details on the number of events were not reported for each patient subgroup, with only the hazard ratios being available. As a matter of fact, all-cause death is an endpoint affected by the influence of many factors linked to the composition and characteristics of the population that may influence the outcome, and the main clinical interest should be focused on end points more strictly linked to AF, such as stroke and thromboembolism. Indeed, randomized studies on AF management were not planned to primarily influence all-cause death, but were rather addressed to reduce thromboembolic events, and the clinical questions related to asymptomatic AF should primarily target thromboembolism and stroke.

### 4.2. Importance of Integrated Approach to AF Care

Moreover, our metanalysis found no differences between asymptomatic and symptomatic patients with regard to the population profile in terms of age and thromboembolic risk. This appears important in interpreting our main findings because clinical decision making, according to guideline recommendations [1,33,34], is based on clinical considerations that include age and the other components of the CHA_2_DS_2_VASc score. This has important implications given the increasing detection of (often asymptomatic) AF with cardiac devices [9,35,36,37].

Additionally, meta-regression found that some comorbidities contribute to heterogeneity, reflecting different protocols and enrolling criteria. The evaluation of heterogeneity has important limitations because information about comorbidities in the patient population was variable among the studies and sometimes lacking, particularly for chronic kidney disease, a comorbidity that may importantly condition the risk of death and thromboembolic events [38,39].

Given the impact on adverse clinical outcomes other than stroke/TE, the management of AF has evolved towards a more holistic or integrated approach to AF care, as recommended in the guidelines [1,40]. Indeed, compliance with the ABC (atrial fibrillation better care) pathway has been associated with a reduction in mortality, stroke, and major bleeding, as well as hospitalisations [41,42].

Our results suggest that AF patients need to be appropriately treated irrespective of symptomatic presentation, focusing on their profile in terms of thromboembolic risk and comorbidities, as well as therapy adherence [1,43,44,45,46]. This updated metanalysis further outlines the risk of stroke/TE in asymptomatic patients, highlighting the importance of an adequate anticoagulation in high-risk patients, independently of the presence/absence of symptoms.

### 4.3. Role of AF Screening 

Indeed, as asymptomatic and symptomatic AF patients share similar outcomes, considering that the first presentation of a silent AF could be an ischemic stroke [15] and the high prevalence of asymptomatic presentations [21], the role of the screening for early AF detection appears crucial, especially in high-risk groups [15,47]. The rationale for supporting AF screening is that asymptomatic AF may be present in the community, but, being underdiagnosed and undertreated, may expose patients to the risk of thromboembolic events and associated AF-related complications. According to guidelines and consensus documents, AF screening, by pulse palpation or using devices targeting AF detection through specific sensors or electrocardiographic recording, is recommended [1,15,47], although an integrated approach, also considering the emerging role of wearables, is needed [48,49,50]. Up to now, the efficacy of screening in reducing stroke has not been supported by randomized trials, and this raised some caution and the call for more direct evidence [51,52,53].

Indirectly, there is evidence of a benefit in the long term, through the institution of oral anticoagulation in the case of AF detection [54,55], as well as evidence that the risk of stroke is similar in asymptomatic AF with incidental detection in primary care as compared with incident AF presenting clinically in general practice or hospital care [56]. Different systematic and opportunistic screening programs have been proposed [1,15]. Moreover, systematic screening is addressed to the target population, typically those 65 years or older or those at higher risk of developing AF based on predictive scores, such as C_2_HEST [57,58]. Opportunistic screening by pulse palpation or ECG rhythm strip during a medical visit for any reason is recommended by the most recent guidelines and consensus documents in older patients (≥65 years old) [1,15]. Handheld devices with ECG capabilities may help in AF screening [59], but a role for wearables and ‘smart’ technology is emerging [39,60,61].

### 4.4. Limitations 

Some limitations to our study should be acknowledged. The main limitation is the moderate–high heterogeneity between studies considered. The variability of our data reflects the observational nature of the majority of studies included, and the retrospective characteristics of some studies. Moreover, we had no access to individual data. We have to consider other limitations related to differences in the methods for assessing symptoms, in mean follow-up time, in the type of anticoagulant used, and persistence and adherence to treatments for AF management. Moreover, the definition of TE as an end point showed some difference across the various studies.

## 5. Conclusions

Mortality and stroke/TE events in AF patients were unrelated to symptomatic status of their clinical presentation. Adoption of management strategies in AF patients should not be based on symptomatic clinical status.

## Figures and Tables

**Table 1:** Studies design and baseline characteristics of asymptomatic and symptomatic AF patients.

Study (Year)	Study Design	Asymptomatic AF						Symptomatic AF						FU (Years)	Outcomes
		N	Age (Mean/Median)	Male *n* (%)	DM *n* (%)	HF *n* (%)	CAD *n* (%)	N	Age (Mean/Median)	Male *n* (%)	DM *n* (%)	HF *n* (%)	CAD *n* (%)		
Flaker GC. [19] (2005)	RCT	481	70 ± 8.3	370 (76.9)	100 (21)	64 (13)	137 (28)	3576	69.7 ± 9	2095 (58.5)	713 (20)	873 (24)	1413 (40)	3.5 (mean)	All cause death, stroke
Komatsu [29] T. (2010)	Retrospective	45	67 ± 9.9	35 (77.7)	14 (31)	NA	NA	289	66.6 ± 11.8	194 (67.1)	28 (10)	NA	NA	5 ± 0.9	CV death, TE event
Potpara TS. [21] (2013)	Prospective	146	53.1 ± 13	122 (83.5)	17 (11.6)	12 (8.2)	7 (4.8)	954	52.6 ± 12.1	589 (61.7)	59 (6.2)	71 (7.4)	46 (4.8)	9.9 ± 6.1	All cause death, CV death, TE events, stroke
Rienstra M. [17] (2014)	RCT	157	67 ± 9	113 (71.9)	14 (9)	37 (34)	25 (16)	365	69 ± 9	217 (67.7)	39 (11)	224 (61)	118 (32)	2.3 ± 0.6	CV death, TE events
Senoo K. [23] (2014)	Retrospective	468	61.2 ± 12.6	373 (79.7)	48 (10.3)	NA	36 (7.7)	708	61.5 ± 13.5	502 (71)	85 (12)	NA	48 (6.8)	3.3 ± 2.5	All cause death, CV death, stroke
Boriani G. [24] (2015)	Prospective	1237	72 (64–78)	804 (65)	276 (22.3)	544 (44.3)	496 (40.1)	1882	68 (61–76)	1056 (56.1)	365 (19.4)	933 (49.6)	636 (33.8)	1 ± 0.1	All cause death, TE events
Bakhai A. [30] (2016)	Prospective	501	71.5 ± 9.3	387 (77.2)	101 (20.2)	44 (8.8)	80 (16)	5695	71.8 ± 10.4	3347 (58.8)	1267 (22.3)	1290 (22.7)	1237 (24.4)	1	Stroke
Guerra F. [31] (2017)	Prospective	AS 252AA 903	AS 67.6 ± 11.4 AA 68.1 ± 11.7	AS 157 (62.3) AA 600 (66.4)	AS 50 (19.9) AA 184 (20.4)	AS 57 (22.7) AA 203 (22.6)	AS 7 (2.8) AA 150 (16.7)	SS 896 SA 1556	SS 65.9 ± 11.6 SA 64.4 ± 13.6	SS 497 (55.5) SA 872 (56)	SS 164 (18.4) SA 244 (15.7)	SS 357 (40.2) SA 418 (27)	SS 134 (15.1) SA 181 (11.7)	1	TE events, stroke
Thind M. [22] (2018)	Prospective	3582	76 (68–82)	2313 (64.6)	1061 (29.6)	1011 (28.2)	1292 (36.1)	5737	74 (66–81)	3062 (53.4)	1686 (29.4)	1958 (34.1)	2041 (35.6)	2.6	All cause death
Gibbs H. [20] (2021)	Prospective	13,235	72 (65–79)	8501 (64.2)	3037 (23)	1786 (13.5)	1219 (9.2)	38797	70 (62–78)	20,541 (52.9)	8509 (21.9)	9953 (25.7)	4317 (11.2)	2	All cause death, TE events

Legend: AF, atrial fibrillation; AS: asymptomatic at baseline symptomatic at 1-year follow-up, AA: asymptomatic at baseline and after 1-year follow-up; CAD, coronary artery disease; CV, cardiovascular; DM, diabetes mellitus; HF, heart failure; NA: not available; RCT, randomized controlled trial; SS: symptomatic at baseline and at 1-year follow-up, SA: symptomatic at baseline and asymptomatic at 1-year follow-up, TE: thromboembolic events, FU: follow-up.

**Table 2:** Thromboembolic risk and anti-thrombotic treatment in asymptomatic and symptomatic AF patients.

Study (Year)	Asymptomatic AF					Symptomatic AF				
	N	Thromboembolic Risk		Antithrombotic Treatment *n* (%)		N	Thromboembolic Risk		Antithrombotic Treatment *n* (%)	
Flaker GC. [19] (2005)	481	NA		Aspirin 100 (21) Warfarin 438 (91)		3576	NA		Aspirin 980 (27) Warfarin 2993 (84)	
Komatsu T. [29] (2010)	45	CHADS_2_ (mean): 1.63 ± 1.27		Aspirin 19 (42) Warfarin 11 (24) None 15 (34)		289	CHADS_2_ (mean): 1.14 ± 1.18		Aspirin 80 (28) Warfarin 66 (23) None 143 (49)	
Potpara TS. [21] (2013)	146	CHADS_2_ ≥ 2, 21 (14.4) CHA_2_DS_2_-VASC ≥ 2, 48 (32.9)		Aspirin 70 (47.9) OAC 59 (40.4) None 17 (11.6)		954	CHADS_2_ ≥ 2, 96 (10.1) CHA_2_DS_2_-VASC ≥ 2, 348 (36.5)		Aspirin 463 (48.6) OAC 203 (21.3) None 287 (30.1)	
Rienstra M. [17] (2014)	157	CHADS_2_ (mean): 1.2 ± 1.1		NA		365	CHADS_2_ (mean): 1.7 ± 1.1		NA	
Senoo K. [23] (2014)	468	CHADS_2_ ≥ 2, 98 (20.9)		Antiplatelets 177 (40.9) Warfarin 150 (32.1) Dabigatran 8 (1.7)		708	CHADS_2_ ≥ 2, 172 (24.3)		Antiplatelets 291 (39.2) Warfarin 234 (33.1) Dabigatran 66 (9.3)	
Boriani G. [24] (2015)	1237	CHADS_2_ (mean): 2 ± 1.31 CHA_2_DS_2_-VASC (mean): 3.41 ± 1.78		Antiplatelets 400 (32.3)OAC 1027 (83) None 45 (3.6)		1882	CHADS_2_ (mean): 1.87 ± 1.25 CHA_2_DS_2_-VASC (mean): 3.14 ± 1.79		Antiplatelets 668 (35.5) OAC 1528 (81.2) None 105 (5.6)	
Bakhai A. [30] (2016)	501	CHA_2_DS_2_-VASC (mean): 2.9 ± 1.7		Antiplatelets 55 (11) VKA 359 (71.7) NOAC 26 (5.2) None 28 (5.6)		5695	CHA_2_DS_2_-VASC (mean): 3.4 ± 1.8		Antiplatelets 634 (11.1) VKA 3781 (66.4) NOAC 359 (6.3) None 336 (5.9)	
Guerra F. [31] (2017)	AS 252AA 903	AS CHADS_2_ (mean): 1.5 ± 1CHA_2_DS_2_-VASC (mean): 2.9 ± 1.8	AA CHADS_2_ (mean): 1.5 ± 1CHA_2_DS_2_-VASC (mean): 2.9 ± 1.7	AS Antiplatelets 73 (29.4) OAC 144 (58.8)	AA Antiplatelets 258 (28.9) OAC 529 (59.8)	SS 896SA 1556	SS CHADS_2_(mean): 1.7 ± 1 CHA_2_DS_2_-VASC (mean): 3.2 ± 2	SA CHADS_2_(mean): 1.4 ± 1 CHA_2_DS_2_-VASC (mean): 2.7 ± 1.8	SS Antiplatelets 285 (32.2) OAC 637(72.4)	SA Antiplatelets 508 (32.9) OAC 964 (63.8)
Thind M. [22] (2018)	3582	CHADS_2_: 2 (1–3) CHA_2_DS_2_-VASC: 4 (3–5)		Antiplatelets 1662 (46.4) OAC 2762 (77.1)		5737	CHADS_2_: 2 (1–3) CHA_2_DS_2_-VASC: 4 (3–5)		Antiplatelets 2767 (48.2) OAC 4292 (74.8)	
Gibbs H. [20] (2021)	13,235	CHA_2_DS_2_-VASC: 3 (2–4)		Antiplatelets 2350 (17.9) NOAC ± antiplatelets 3906 (29.8) VKA ± antiplatelets 5187 (39.6) None 1659 (12.7)		38,797	CHA_2_DS_2_-VASC: 3 (2–4)		Antiplatelets 8411 (22) NOAC ± antiplatelets 10,217 (26.7) VKA ± antiplatelets 14,996 (39.3)None 4581 (12)	

Legend: AF, atrial fibrillation; AS: asymptomatic at baseline symptomatic at 1-year follow-up, AA: asymptomatic at baseline and after 1-year follow-up; NA: not available, NOAC: new oral anticoagulants; OAC: oral anticoagulants; SS: symptomatic at baseline and at 1-year follow-up, SA: symptomatic at baseline and asymptomatic at 1-year follow-up; VKA: vitamin K antagonists; CHADS_2:_ Congestive heart failure, Hypertension, Age, Diabetes, previous Stroke and/or TIA (transient ischemic attack); CHA2DS2-VASC: congestive heart failure, hypertension, age, diabetes, previous stroke and/or TIA, vascular disease, female sex category.

## Supplementary Materials

The following are available online at https://www.mdpi.com/article/10.3390/jcm10173979/s1, Table S1: Full search key for the metanalysis with the terms used, Table S2: Antiarrhythmic treatments in asymptomatic and symptomatic AF patients, Table S3: Incidence of type of AF of asymptomatic and symptomatic patients, Table S4: Tools used to stratify symptomatic AF patients, Table S5: Newcastle-Ottawa quality assessment for cohort studies, Table S6: Sensitivity analysis, Table S7: Univariate meta-regression analysis for risk of all-cause death, Table S8: Univariate meta-regression analysis for risk of stroke or systemic embolism. Figure S1: Age differences in asymptomatic and symptomatic AF patients, Figure S2: Panel A: Analysis of the CHA2DS2VASc, Panel B: Analysis of CHADS2, Figure S3: Funnel plots for publication bias (Panel A: All cause death analysis, Panel B: Cardiovascular death analysis, Panel C: TE/stroke analysis, Panel D: Stroke analysis).
