# Supplementary material for: Comparing Outcomes in Asymptomatic and Symptomatic Atrial Fibrillation: A Systematic Review and Meta-Analysis of 81,462 Patients
Source: J Clin Med. 2021 Sep 2;10(17):3979. doi: 10.3390/jcm10173979 (PMC8432492; doi:10.3390/jcm10173979)
Supplement: Supplementary file 1 [file jcm-10-03979-s001.zip › jcm-1351804-supplementary.pdf]

Journal of Clinical Medicine

MDPI

Supplementary materials

Table S1. Full search key for the meta-analysis with the terms used.

Full search
("asymptomatic"[All Fields] OR "asymptomatically"[All Fields] OR "asymptomatics"[All Fields] OR ("symptomatic"[All Fields] OR "symptomatically"[All Fields] OR "symptomat-ics"[All Fields])) AND "atrial fibrillation"[All Fields] AND ("outcome"[All Fields] OR "out-comes"[All Fields] OR ("death"[MeSH Terms] OR "death"[All Fields] OR "deaths"[All Fields]) OR ("mortality"[MeSH Terms] OR "mortality"[All Fields] OR "mortalities"[All Fields] OR "mortality"[MeSH Subheading]) OR ("stroke"[MeSH Terms] OR "stroke"[All Fields] OR "strokes"[All Fields] OR "stroke s"[All Fields]) OR ("thromboembolic"[All Fields] OR "thromboembolism"[MeSH Terms] OR "thromboembolism"[All Fields] OR "thromboembo-lisms"[All Fields] OR "thromboembolization"[All Fields]) OR ("embol"[All Fields] OR "embolics"[All Fields] OR "embolisations"[All Fields] OR "embolise"[All Fields] OR "embo-lised"[All Fields] OR "embolising"[All Fields] OR "embolism"[MeSH Terms] OR "embo-lism"[All Fields] OR "embolic"[All Fields] OR "embolisms"[All Fields] OR "embolization, therapeutic"[MeSH Terms] OR ("embolization"[All Fields] AND "therapeutic"[All Fields]) OR "therapeutic embolization"[All Fields] OR "embolisation"[All Fields] OR "embolization"[All Fields] OR "embolizations"[All Fields] OR "embolize"[All Fields] OR "embolized"[All Fields] OR "embolizes"[All Fields] OR "embolizing"[All Fields]))

Table S2. Anti-arrhythmic treatments in asymptomatic and symptomatic AF patients.

Study	Asymptomatic AF				Symptomatic AF			
	N	AADs *n* (%)	CatAbl *n* (%)	BB *n* (%)	N	AADs *n* (%)	CatAbl *n* (%)	BB *n* (%)
Flaker GC. (2005)	481	NA	NA	185 (39)	3576	NA	NA	1541 (43)
Komatsu T. (2010)	45	NA	NA	NA	289	NA	NA	NA
Potpara TS. (2013)	146	Ia/Ic/ Sotalol 16 (11)<br>Amiodarone 33 (22.6)<br>None 18 (12.3)	NA	NA	954	Ia/Ic/ Sotalol 140 (14.7)<br>Amiodarone 119 (12.5)<br>None 161 (16.9)	NA	NA
Rienstra M. (2014)	157	NA	NA	NA	365	NA	NA	NA
Senoo K. (2014)	468	Class Ia/Ic 205 (43.8)	NA	130 (27.8)	708	Class Ia/Ic 317 (44.8)	NA	229 (32.3)
Boriani G. (2015)	1237	At least one 327 (26.4)<br>Amiodarone 161 (13)	74 (6)	861 (69.6)	1882	At least one 787 (41.8)<br>Amiodarone 502 (26.7)	201 (10.7)	1304 (69.3)
Bakhai A. (2016)	501	Amiodarone 72 (14.4)<br>Flecainide 27 (5.4)<br>Propafenone 8 (1.6)<br>Sotalol 28 (5.6)	NA	NA	5695	Amiodarone 1417 (24.9)<br>Flecainide 596 (10.5)<br>Propafenone 178 (3.1)<br>Sotalol 318 (5.6)	NA	NA
Guerra F. (2017)	AS 252	<u>AS</u><br>Ia 2 (0.8)	AS 6 (2.4)<br>AA 8 (0.9)	AS 41 (16.7)	SS 896	<u>SS</u><br>Ia 3 (0.3)	SS 39 (4.4)<br>SA 53 (3.4)	SS 114 (12.9)<br>SA 223 (14.7)

	AA 903	Ic 14 (5.6)<br>Class III 59 (23.8)<br><u>AA</u><br>Ia 2 (0.2)<br>Ic 52 (5.8)<br>Class III 240 (26.8)		AA 183 (20.7)	SA 1556	Ic 120 (13.5)<br>Class III 309 (34.9)<br><u>SA</u><br>Ia 10 (0.6)<br>Ic 173 (11.2)<br>Class III 529 (34.3)		
Thind M. (2018)	3582	802 (22.4)	NA	NA	5737	1900 (33.1)	NA	NA
Gibbs H. (2021)	13,235	NA	NA	NA	38,797	NA	NA	NA

AADs, antiarrhythmics drugs, AS: asymptomatic at baseline symptomatic at 1-year follow-up, AA: asymptomatic at baseline and after 1-year follow-up, BB, beta-blockers; CatAbl: catheter ablation; NA: not available; SS: symptomatic at baseline and at 1-year follow-up, SA: symptomatic at baseline and asymptomatic at 1-year follow-up.

Table S3. Incidence of type of AF in asymptomatic and symptomatic patients.

Study	Asymptomatic AF					Symptomatic AF				
	N	New onset *n* (%)	Paroxysmal *n* (%)	Persistent *n* (%)	Permanent *n* (%)	N	New onset *n* (%)	Paroxysmal *n* (%)	Persistent *n* (%)	Permanent *n* (%)
Flaker GC. (2005)	481	170 (35,3)	NA	NA	NA	3576	1221 (34,1)	NA	NA	NA
Komatsu T. (2010)	45	-	45 (100)	-	-	289	-	289 (100)	-	-
Potpara TS. (2013)	146	-	39 (26.7)	40 (27.4)	70 (45.9)	954	-	626 (65.6)	185 (19.4)	143(15)
Rienstra M. (2014)	157	-	-	157 (100)	-	365	-	-	365 (100)	-
Senoo K. (2014)	468	-	468 (100)	-	-	708	-	708 (100)	-	-
Boriani G. (2015)	1237	388 (31.4)	290 (23.5)	203 (16.4)	356 (28.8)	1882	557 (29.6)	536 (28.5)	606 (32.2)	183 (9.7)
Bakhai A. (2016)	501	-	112/498 (22.5)	158/498 (31.7)	228/498 (45.8)	5695	-	1714/5686 (30.1)	1773/5686 (31.2)	2199/5686 (38.7)
Guerra F. (2017)	AS 252<br>AA 903	AS 18 (7.1)<br>AA 139 (15.4)	AS 67 (26.6)<br>AA 175 (19.4)	AS 50 (20)<br>AA 181 (20)	AS 102 (40.5)<br>AA 378 (41.9)	SS 896<br>SA 1556	SS 74 (8.2)<br>SA 404 (25.9)	SS 281 (31.4)<br>SA 494 (31.7)	SS 233 (26)<br>SA 357 (22.9)	SS 301 (33.6)<br>SA 280 (18)
Thind M. (2018)	3582	120 (3.3)	1643 (45.8)	1819 (50.8)		5737	306 (5.3)	3134 (54.6)	2297 (40)	

Gibbs H. (2021)	13,235	5643 (42.6)	3324 (25.1)	2087 (15.7)	2176 (16.4)	38,797	17,688 (45.6)	10,983 (28.3)	5671 (14.6)	4454 (11.5)

NA, not available.

Table S4. Tools used to stratify symptomatic AF patients.

Study (year)	Tool	Symptoms evaluated
**Flaker GC. (2005)**	**15-item check list (at least one to define symptomatic AF patients)**	Arrhythmic symptoms: dizziness, light-headedness, fast heartbeat, palpitations, or syncope. Heart failure symptoms: dyspnea, edema, orthopnea, or paroxysmal nocturnal dyspnea. A third group: chest pain, diaphoresis, diuresis, fatigue, or panic.
**Komatsu T. (2010)**	**NA**	Arrhythmic symptoms: dizziness, light-headedness, fast heartbeat, palpitations, or syncope. Heart failure symptoms: dyspnea, edema, orthopnea, or paroxysmal nocturnal dyspnea.
**Potpara TS. (2013)**	**NA**	Palpitations, tachycardia, fatigue, malaise, shortness of breath on exertion, dyspnoea, chest pain, syncope or presyncope, or worsening of pre-existent symptoms related to other illness.
**Rienstra M. (2014)**	**7-item check list (at least one to define symptomatic AF patients)**	Palpitation, fatigue, dizziness, dyspnoea, chest pain, or other symptoms.
**Senoo K. (2014)**	**NA**	Palpitations, tachycardia, fatigue, malaise, shortness of breath on exertion, dyspnea, chest pain, syncope, or presyncope.
**Boriani G. (2015)**	**EHRA score**	Palpitation, fatigue, dizziness, dyspnoea, chest pain, or syncope.
**Bakhai A. (2016)**	**EHRA score**	Palpitation, fatigue, dizziness, dyspnoea, chest pain, or syncope.
**Guerra F. (2017)**	**NA**	One or more of the following symptoms: palpitations, syncope, dyspnea, chest pain, dizziness, and fatigue.
**Thind M. (2018)**	**EHRA score**	Palpitation, fatigue, dizziness, dyspnoea, chest pain, or syncope.
**Gibbs H. (2021)**	**NA**	At least one of the following clinical features documented at baseline: palpitations, shortness of breath, chest pain/discomfort, dizziness, tiredness, sweating, or fainting, and those with signs such as irregular pulse or tachycardia.

NA, not available.

Table S5. Newcastle–Ottawa quality assessment for cohort studies.

Study,year	Selection	Comparability	Outcome	Total
Komatsu T., 2010	***	**	***	8
Potpara T. S., 2013	****	**	***	9
Senoo K., 2014	***	**	***	8
Boriani G., 2015	***	**	***	8
Bakhai A., 2016	***	-	***	6
Guerra F., 2017	***	**	***	8
Thind M., 2018	***	**	***	8
Gibbs H., 2021	***	**	***	8

Table S6. Sensitivity analysis.

Study	Pooled risk ratio (95% CI)	Heterogeneity I^2	Heterogeneity *p*-value
**All-cause death**			
**Pooled analysis (all included)**	**1.03 (0.81–1.32)**	**86%**	***p* < 0.00001**
Remove: Flaker 2005	1.13 (0.86–1.48)	87%	*p* < 0.00001
Remove: Potpara 2013	1.05 (0.81–1.37)	89%	*p* < 0.00001
Remove: Senoo 2014	1.05 (0.81–0.36)	89%	*p* < 0.00001
Remove: Boriani 2015	0.93 (0.86–1.00)	10%	*p* = 0.35
Remove: Thind 2018	1.05 (0.70–1.60)	89%	*p* < 0.00001
Remove: Gibbs 2021	1.05 (0.67–1.63)	89%	*p* < 0.00001
**TE/stroke**			
**Pooled analysis (all included**)	**1.06 (0.86–1.31)**	**49%**	***p* = 0.04**
Remove: Flaker 2005	1.06 (0.84–1.34)	53%	*p* = 0.03
Remove: Komatsu 2010	0.99 (0.83–1.18)	31%	*p* = 0.17
Remove: Potpara 2013	1.01 (0.82–1.24)	43%	*p* = 0.08
Remove: Rienstra 2014	1.09 (0.87–1.36)	54%	*p* = 0.03
Remove: Senoo 2014	1.10 (0.89–1.36)	48%	*p* = 0.05
Remove: Boriani 2015	1.07 (0.85–1.34)	54%	*p* = 0.02
Remove: Bakhai 2016	1.01 (0.83–1.23)	41%	*p* = 0.10
Remove: Guerra 2017	1.08 (0.85–1.38)	54%	*p* = 0.02
Remove: Thind 2018	1.11 (0.86–1.45)	54%	*p* = 0.03
Remove: Gibbs 2021	1.13 (0.87–1.47)	47%	*p* = 0.06
**Stroke**			
**Pooled analysis (all included**)	**1.22 (0.77–1.93)**	**62%**	***p* = 0.03**
Remove: Flaker 2005	1.24 (0.66–2.35)	72%	*p* = 0.01
Remove: Potpara 2013	1.06 (0.67–1.67)	53%	*p* = 0.10
Remove: Senoo 2014	1.43 (0.95–2.13)	44%	*p* = 0.15
Remove: Bakhai 2016	1.10 (0.66–1.81)	64%	*p* = 0.04
Remove: Guerra 2017	1.31 (0.74–2.33)	69%	*p* = 0.02

CI, confidence interval; TE, thromboembolic events.

Table S7. Univariate meta-regression analysis for risk of all-cause death.

Moderator	Number of studies	p-value (after regression)	Tau^2	I^2	*p*
Study type	6	0.268	0.065	90.09%	<0.001
Mean age	6	0.802	0.137	86.01%	<0.001
Male sex	6	0.820	0.182	88.65%	<0.001
Diabetes	6	0.810	0.173	88.63%	<0.001
CAD	6	0.732	0.119	88.29%	<0.001
HF	5	0.838	0.191	91.47%	<0.001
OAC	6	0.820	0.180	88.65%	<0.001
Main analysis	6	0.79	0.06	86%	<0.001

CI: confidence interval; CAD: coronary artery disease; HF: heart failure; OAC: oral anticoagulation. The last row shows the results of the main analysis to help the reader in the comparison.

Table S8. Univariate meta-regression analysis for risk of stroke or systemic embolism.

Moderator	Number of studies	*p*-value (after regression)	Tau^2	I^2	*p*
Study type	10	0.959	0.065	58.66%	0.018
Mean age	7	0.791	0.117	57.98%	0.027
Male sex	10	0.386	0.064	45.62%	0.065

Diabetes	10	0.361	0.061	44.93%	0.069
CAD	9	0.384	0.024	28.18%	0.203
HF	7	0.159	0.009	11.86%	0.339
OAC	8	0.328	0.071	49.75%	0.052
Main analysis	10	0.58	0.05	49%	0.04

CI: confidence interval; CAD: coronary artery disease; HF: heart failure; OAC: oral anticoagulation. The last row shows the results of the main analysis to help the reader in the comparison.

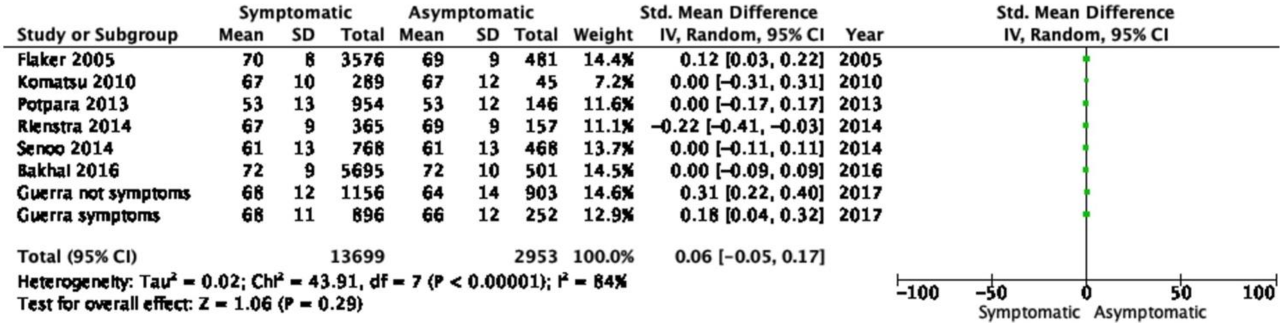

Study or Subgroup	Symptomatic Mean	SD	Total	Asymptomatic Mean	SD	Total	Weight	Std. Mean Difference IV, Random, 95% CI	Year
Flaker 2005	70	8	3576	69	9	481	14.4%	0.12 [0.03, 0.22]	2005
Komatsu 2010	67	10	289	67	12	45	7.2%	0.00 [−0.31, 0.31]	2010
Potpara 2013	53	13	954	53	12	146	11.6%	0.00 [−0.17, 0.17]	2013
Rienstra 2014	67	9	365	69	9	157	11.1%	−0.22 [−0.41, −0.03]	2014
Senoo 2014	61	13	768	61	13	468	13.7%	0.00 [−0.11, 0.11]	2014
Bakhal 2016	72	9	5695	72	10	501	14.5%	0.00 [−0.09, 0.09]	2016
Guerra not symptoms	68	12	1156	64	14	903	14.6%	0.31 [0.22, 0.40]	2017
Guerra symptoms	68	11	896	66	12	252	12.9%	0.18 [0.04, 0.32]	2017
Total (95% CI)			13699			2953	100.0%	0.06 [−0.05, 0.17]	

Heterogeneity: Tau² = 0.02; Chi² = 43.91, df = 7 (P < 0.00001); I² = 84%
Test for overall effect: Z = 1.06 (P = 0.29)

Data from Guerra et al. (30) were divided into two according to the original paper ("*Guerra not symptoms*" indicates patients asymptomatic at follow-up and "*Guerra symptoms*" refers to symptomatic patients at follow-up).

Figure S1. Age differences in asymptomatic and symptomatic AF patients.

Panel A.

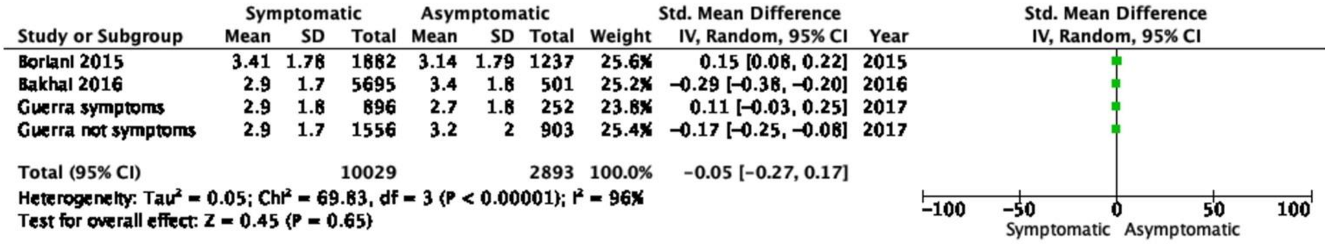

Study or Subgroup	Symptomatic Mean	SD	Total	Asymptomatic Mean	SD	Total	Weight	Std. Mean Difference IV, Random, 95% CI	Year
Borlani 2015	3.41	1.78	1882	3.14	1.79	1237	25.6%	0.15 [0.08, 0.22]	2015
Bakhal 2016	2.9	1.7	5695	3.4	1.8	501	25.2%	−0.29 [−0.38, −0.20]	2016
Guerra symptoms	2.9	1.8	896	2.7	1.8	252	23.8%	0.11 [−0.03, 0.25]	2017
Guerra not symptoms	2.9	1.7	1556	3.2	2	903	25.4%	−0.17 [−0.25, −0.08]	2017
Total (95% CI)			10029			2893	100.0%	−0.05 [−0.27, 0.17]	

Heterogeneity: Tau² = 0.05; Chi² = 69.83, df = 3 (P < 0.00001); I² = 96%
Test for overall effect: Z = 0.45 (P = 0.65)

Panel B.

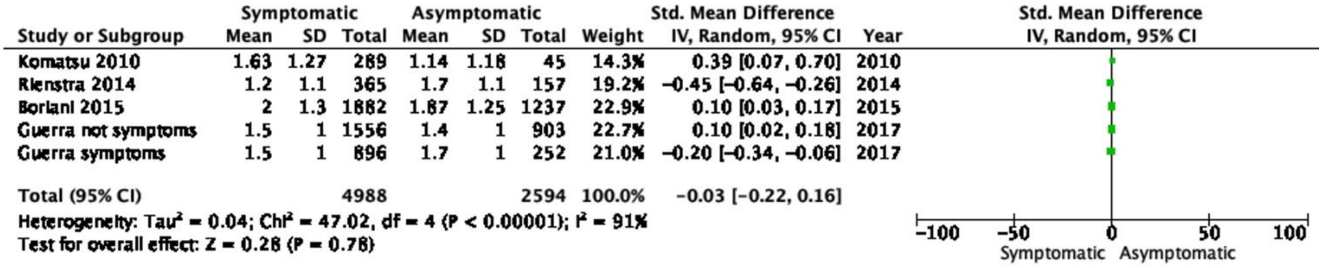

Study or Subgroup	Symptomatic Mean	SD	Total	Asymptomatic Mean	SD	Total	Weight	Std. Mean Difference IV, Random, 95% CI	Year
Komatsu 2010	1.63	1.27	289	1.14	1.18	45	14.3%	0.39 [0.07, 0.70]	2010
Rienstra 2014	1.2	1.1	365	1.7	1.1	157	19.2%	−0.45 [−0.64, −0.26]	2014
Borlani 2015	2	1.3	1882	1.87	1.25	1237	22.9%	0.10 [0.03, 0.17]	2015
Guerra not symptoms	1.5	1	1556	1.4	1	903	22.7%	0.10 [0.02, 0.18]	2017
Guerra symptoms	1.5	1	896	1.7	1	252	21.0%	−0.20 [−0.34, −0.06]	2017
Total (95% CI)			4988			2594	100.0%	−0.03 [−0.22, 0.16]	

Heterogeneity: Tau² = 0.04; Chi² = 47.02, df = 4 (P < 0.00001); I² = 91%
Test for overall effect: Z = 0.28 (P = 0.78)

Figure S2. Panel A: Analysis of the CHA_2DS_2VASc. Panel B: Analysis of $CHADS_2$.

Panel A.

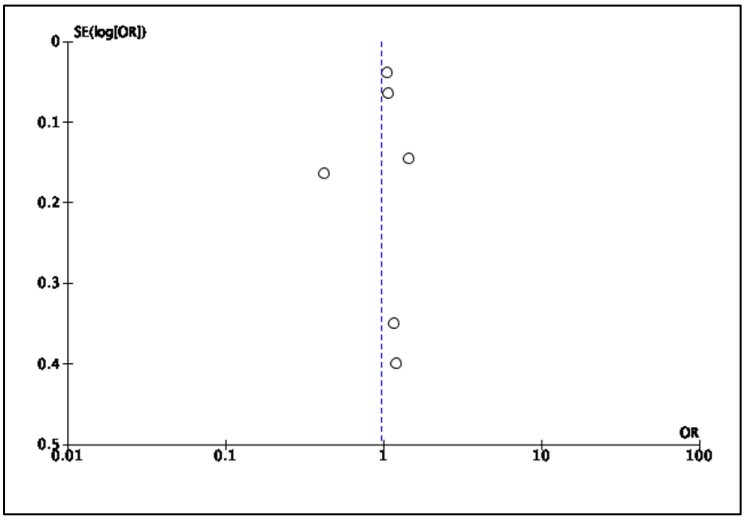

Panel B.

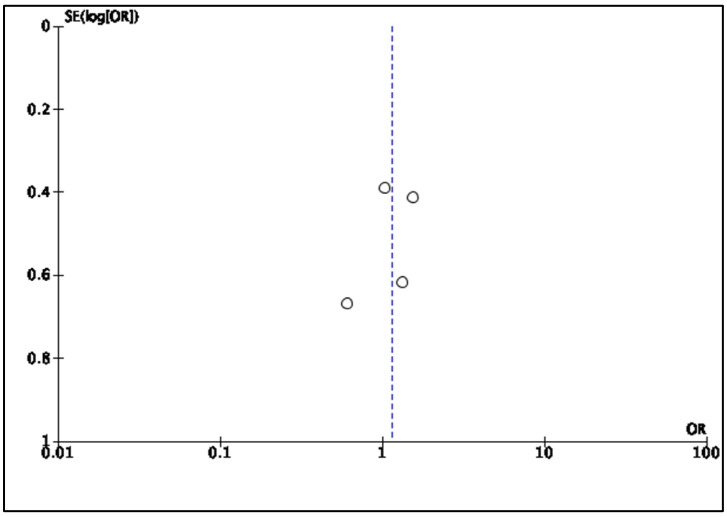

Panel C.

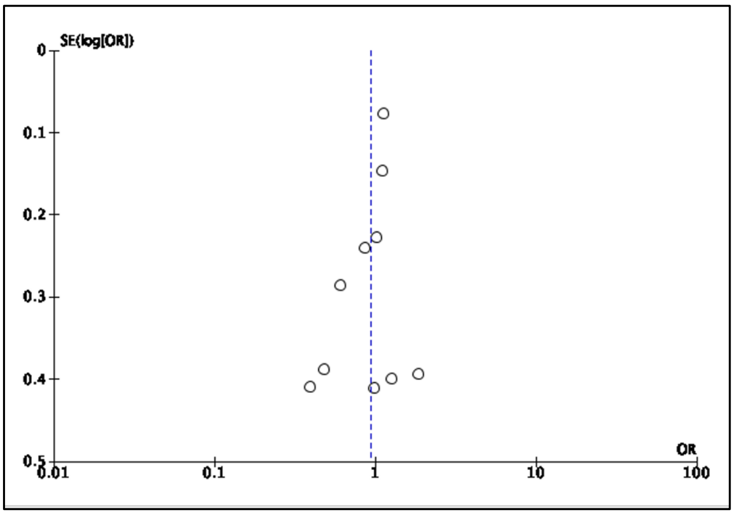

Panel D.

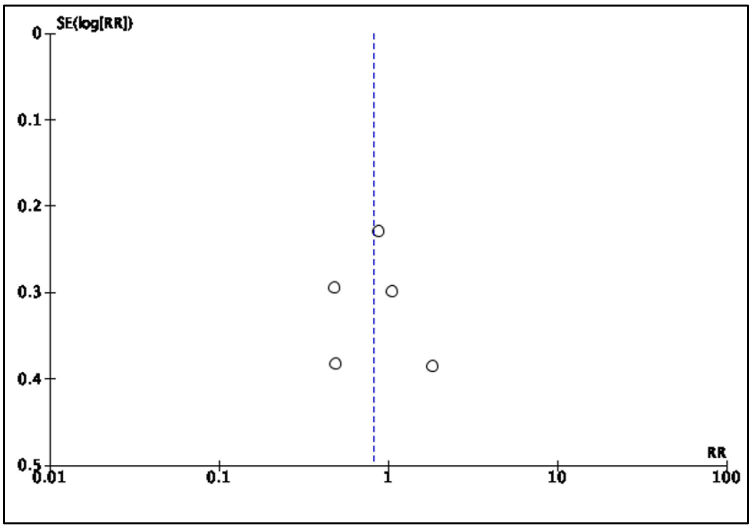

Figure S3. Funnel plots for publication bias ((A) All-cause death analysis. (B) Cardiovascular death analysis. (C) TE/stroke analysis. (D) Stroke analysis).
